# Cytological Approaches Combined With Chemical Analysis Reveals the Layered Nature of Flax Mucilage

**DOI:** 10.3389/fpls.2019.00684

**Published:** 2019-06-21

**Authors:** Fabien Miart, Françoise Fournet, Nelly Dubrulle, Emmanuel Petit, Hervé Demailly, Loic Dupont, Luciane Zabijak, Paulo Marcelo, Arezki Boudaoud, Christophe Pineau, Stéphanie Guénin, Olivier Van Wuytswinkel, François Mesnard, Karine Pageau

**Affiliations:** ^1^Unité Biologie des Plantes et Innovation, EA-3900, Université de Picardie Jules Verne, UFR des Sciences, Amiens, France; ^2^Reproduction et Développement des Plantes, Université de Lyon, École Normale Supérieure de Lyon, Université Claude Bernard Lyon 1, Institut National de la Recherche Agronomique, Centre National de la Recherche Scientifique, Lyon, France; ^3^Centre de Ressources Régionales en Biologie Moléculaire, UFR des Sciences, Amiens, France; ^4^Laboratoire de Réactivité et de Chimie des Solides, CNRS UMR 7314, Université de Picardie Jules Verne, UFR des Sciences, Amiens, France; ^5^Plateforme d’Ingénierie Cellulaire et d’Analyses des Protéines, Centre Universitaire de Recherche en Santé, Amiens, France

**Keywords:** cell wall, flax, mucilage, polysaccharide layers, peeling mechanism, AX/RG I ratio

## Abstract

The external seed coat cell layer of certain species is specialized in the production and extrusion of a polysaccharide matrix called mucilage. Variations in the content of the released mucilage have been mainly associated with genetically regulated physiological modifications. Understanding the mucilage extrusion process in crop species is of importance to gain deeper insight into the complex cell wall biosynthesis and dynamics. In this study, we took advantage of the varying polysaccharide composition and the size of the flax mucilage secretory cells (MSCs) to study mucilage composition and extrusion in this species of agricultural interest. We demonstrate herein that flax MSCs are structured in four superimposed layers and that rhamnogalacturonans I (RG I) are firstly synthesized, in the upper face, preceding arabinoxylan and glucan synthesis in MSC lower layers. Our results also reveal that the flax mucilage release originates from inside MSC, between the upper and deeper layers, the latter collaborating to trigger polysaccharide expansion, radial cell wall breaking and mucilage extrusion in a peeling fashion. Here, we provide evidence that the layer organization and polysaccharide composition of the MSCs regulate the mucilage release efficiency like a peeling mechanism. Finally, we propose that flax MSCs may represent an excellent model for further investigations of mucilage biosynthesis and its release.

## Introduction

Recently described as a new model for the study of carbohydrate metabolism and cell wall properties (Arsovski et al., 2010; Haughn and Western, 2012; Francoz et al., 2015), seed coat mucilage research and its applications for commercial use, like in foaming agents, pet food product design or fabrication of nanofibers (Nybroe et al., 2016; Hadad and Goli, 2018; Kaur et al., 2018), are in part hampered by the fact that the relationships between the mucilage secretory cell (MSC) internal organization, the mucilage chemical composition and the events leading to its extrusion are poorly known. Functional analysis of mucilage biosynthesis and extrusion process are mainly focused on Arabidopsis mutants at the expense of other under-exploited genetic resources such as natural variants and other species like *Plantago* (North et al., 2014; Phan et al., 2016; Voiniciuc et al., 2016). The large size of the flax MSC, the growing number of studies on flax mucilage polysaccharide composition and its high industrial applications in various industries (Bhatty, 1993; Westscott and Muir, 2003; Alix et al., 2008; Choudhary and Pawar, 2014; Shim et al., 2014) make the flax seed coat mucilage an attractive model to be investigated.

The endosperm of Arabidopsis is surrounded by four to five cell layers of mature seed coat, the epidermal cell layer of the outer integument composed of MSCs (Western et al., 2000; Windsor et al., 2000; Macquet et al., 2007a). Differentiation of the MSCs and mucilage production (biosynthesis, secretion), composition, regulation and dynamics have been widely detailed in this plant model (North et al., 2014; Creff et al., 2015; Francoz et al., 2015; Voiniciuc et al., 2015b). Arabidopsis MSCs are hexagonal cells with a central volcano-shaped cellulosic structure called the columella, surrounded by a mucilage polysaccharide-containing ring (Voiniciuc et al., 2015b). When hydrated, the seeds quickly release two mucilage layers successively, one usually called “non-adherent” and another one usually called “adherent,” mainly composed of rhamnogalacturonans type I (RG I). RG I has a backbone of alternating (1→2)-α-l-rhamnose (Rha) and (1→4)-β-d-GalA that can be substituted on the Rha residues with side-chains consisting of arabinans, galactans, type I arabinogalactans and terminal galactose (*t*-Gal) residues (Penfield et al., 2001; Macquet et al., 2007a; Haughn and Western, 2012). Once seeds have been soaked in water, mucilage polysaccharides are likely to expand in the MSCs due to their high hydrophilic properties, applying sufficient pressure to cause a rupture between distal and radial primary cell walls to extrude mucilage (Western et al., 2000; North et al., 2014, Francoz et al., 2015). Correct outer cell wall structure requires several gene families as a class III peroxidase, PEROXIDASE 36 (PER36), pectin methylesterase inhibitor (PMEI6), subtilase (SUBT1.7) or transcriptional regulators (LUH1, MUM1)… to allow mucilage release (Kunieda et al., 2013; Ranocha et al., 2014; Francoz et al., 2015). The hydroxy radicals generated degrade polysaccharides in a localized manner, thereby, weakening the outer cell wall (Kunieda et al., 2013).

Studies of Arabidopsis mutants have already correlated mucilage extrusion defects with problems in seed coat differentiation, mucilage biosynthesis, mucilage’s fine chemical composition and structure, swelling and hydration properties (Penfield et al., 2001; Western et al., 2004; Dean et al., 2007; Macquet et al., 2007b; Arsovski et al., 2009; Walker et al., 2011). Modifications in the MSC primary and secondary cell walls (Rautengarten et al., 2008; Kunieda et al., 2013; Saez-Aguayo et al., 2013) and in the composition and structure of the columella have also revealed impaired mucilage extrusion phenotypes (Harpaz-Saad et al., 2011; Mendu et al., 2011; Sullivan et al., 2011). Mucilage biosynthesis and extrusion are regulated mainly through a heavy regulatory network combining TFs (transcription factors) and enzymes involved in polysaccharide metabolism and secretion (North et al., 2014; Francoz et al., 2015; Voiniciuc et al., 2015b).

The structure and polysaccharide composition of flax MSCs largely differ from Arabidopsis (Naran et al., 2008; North et al., 2014), leading to the statement that a detailed study of flax MSCs could represent a possible way to bring new insights into the mucilage release process in this crop. The flax MSC internal structure lacks a mucilage ring (Ozenda, 2000; Attoumbré et al., 2011; Ziolkovska, 2012) and the presence of a columella is still under debate since cellulose staining was found in *Linum strictum* MSCs but not in *Linum usitatissimum* (Western, 2012; North et al., 2014). The physico-chemical behavior of the flax mucilage polysaccharides remains an ongoing debate with regards to the contribution of every class of them in the viscosity and the hydrophilic properties of the mucilage (Cui et al., 1994; Warrand et al., 2005a,b; Paynel et al., 2013; North et al., 2014). Flax seed contains 0.4–10.2% mucilage (Diederichsen et al., 2006). The flax mucilage is composed of RG I with a small amount of homogalacturonans (HGAs) and contains an important fraction of highly branched arabinoxylans (AXs) (Warrand et al., 2005a,b; Naran et al., 2008; Paynel et al., 2013; Ray et al., 2013; Hu et al., 2016a,b). The AXs contain double branches of non-reducing terminal L-arabinosyl units at the O-2 and O-3 positions along the xylan backbone (Naran et al., 2008). In Arabidopsis, xylan chains attached to RG-I allow the adsorption of mucilage to cellulose microfibrils and thus the formation of the adherent mucilage layer (Ralet et al., 2016). The majority of flax RG-I corresponds to RG-I with single non-reducing terminal L-Fuc and L-Gal residues attached to the O-3 position instead of the typical arabinan and galactans (Naran et al., 2008). The backbone of RG-I is composed of diglycosyl repeat units with the following structure →2)-α-L-Rhap-(1→4)-α-D-GalpA-(1→. The ratio of neutral to acidic polysaccharides, i.e., AX/RG I ratio, varies between 0.3 and 2.2 according to the genotype, extraction protocol and environmental conditions (Diederichsen et al., 2006; Western, 2012; Ziolkovska, 2012) and ranges from 75/25% (Cui et al., 1994; Fedeniuk and Biliaderis, 1994; Warrand et al., 2003, 2005a,b) to 27/63% (Paynel et al., 2013), the percentage of the two main polysaccharides RG-I and AX estimated as (2Rha + Fuc + Gal) and (Ara + Xyl), respectively. The flax mucilage is also composed of a third fraction corresponding to a mixture of AXs and RG-I (Naran et al., 2008). While AXs and RG I have a low and high viscosity, respectively, the mixture shows enhanced viscosity, superior to RG I (Naran et al., 2008). In flax mucilage, L-Gal residue is associated with RG-I and D-Gal residue is associated with AXs. The mucilage of flax also has a low protein content at a level of 1–2%.

Unlike Arabidopsis, the non-adherent mucilage layer in flax has condensed mucilage and the adherent mucilage layer is almost non-existent probably reflecting a different composition, a different structure and/or a different mucilage release (Naran et al., 2008). Thus, it is of interest to investigate how the flax MSCs are organized and find out whether the structure and composition of the flax MSCs can be related to the mucilage release process. An analysis of the fine structure of flax MSCs during seed development, combined with the analysis of the chemical composition of the mucilage, should lead to a better understanding of the release process of flaxseed mucilage.

## Materials and Methods

### Plant Material and Growth Conditions

Seeds from the parental cultivars Oliver, winter linseed (producing oil) and Viking, spring fiber flax and the recombinant inbred lines (RILs) from the cross of these two varieties were kindly provided by INRA, Versailles, France. The RILs provided in F6 were selfed to generation F8 as single plants in the green house and then multiplicated in pool in the field (generations F9, F10, F11, and F12). The RILs were obtained from GIE LINEA Semences de Lin (France). Seeds from RIL 44, RIL 283, and RIL 80 were then grown on soil in a greenhouse at 21°C/15°C with a 16 h photoperiod and 55% humidity. For cytological and histological analyses, flowers were tagged during the plant growth and developing seeds removed at the different stages of development.

### Cytochemical Probes

Visualization of the *in vivo* mucilage release and peeling mechanisms on flax mature seeds was carried out by placing seeds in 0.01% (w/v) toluidine blue O (Bergeron and Singer, 1958; Merk) or 0.02% (w/v) ruthenium red (Hanke and Northcote, 1975; Sigma-Aldrich, St Quentin Fallavier, France) in water without shaking. Seeds were examined under bright-field optics with a light microscope (Eclipse 90i, Nikon^1^) and a stereoscopic microscope (SteREO Discovery V20, CARL ZEISS, Germany) depending on the desired resolution. For *in situ* analyses, fixed sections were dipped in a 0.05% (w/v) toluidine blue O or a 0.02% (w/v) ruthenium red bath prior to washing with absolute ethanol and placed on a heating plate for 2 min to improve sample penetration. For confocal microscopy, staining with 0.5% calcofluor white (fluorescent brightener 28, Sigma-Aldrich) was carried out at room temperature for 10 min.

### Cytological Analysis

Seeds were excised from at least two different capsules for each stage of development. Seed tissues were cut transversally and immediately fixed in 4% (w/v) paraformaldehyde containing a 0.1 M sodium phosphate buffer (pH 7.4) to which was added 1% (w/v) saccharose and 0.05% (v/v) Tween 20. Fixation of the seeds was performed with two successive infiltration steps of 15 min under 25 kPa at room temperature. Paraformaldehyde was then removed from samples by successive ethanol baths from 30 to 90% for 30 min under shaking conditions followed by 2 h in 100% ethanol. Seed samples were then included by carefully placing them in 100% EtOH/LR White (v/v) for 5 h followed by pure medium grad acrylic LR White resin (Agar Scientific^2^) for 2 days, taking care to replace the resin every day. Half-seeds were finally placed in capsules, making sure that they did not touch the sides, and placed in a heat chamber for 24 h. Polymerized samples were cut using an ultramicrotome (Leica ultracut UCT) and 2 μm sections were collected on poly-L-lysine-treated well glass slides.

### Immunolocalization

The excess seed sections used for cytological analysis were selected and the immunolabeling was carried out according to Turbant et al. (2016). Primary monoclonal antibodies LM11 and LM15 were retrieved from PlantProbes^3^ (University of Leeds, Leeds) and primary monoclonal antibodies CCRC-M36, CCRC-M141, and CCRC-M58 from the Complex Carbohydrates Research Center^4^ (Carbosource, Athens, GA, United States).

The LM11 monoclonal antibodies were shown to recognize unsubstituted and relatively low-substituted xylans in several species and also more extensive substitution of a xylan backbone. In addition, they were shown to bind strongly to wheat AX (McCartney et al., 2005). The LM15 monoclonal antibodies were shown to recognize xyloglucan of plant cell wall in several species (Marcus et al., 2008). The CCRC-M36 monoclonal antibodies recognize the backbone of RG-I with the requirement of at least three unbranched disaccharide repeats for the antibody to bind; the CCRC-M141 monoclonal antibodies bind to flax mucilage only and the CCRC-M58 monoclonal antibodies are known to recognize xyloglucan (Pattathil et al., 2010).

### Confocal Microscopy

Samples were imaged with a confocal laser microscope (LSM 780, Carl Zeiss). Images were acquired with a ×40 HCX PL APO CS 1.25 NA oil objective with the following parameters: image dimension (512 × 512), scanning speed (400 Hz), line average of 8, pinhole (1 airy unit). Laser power and gain settings for each PMT were slightly adjusted individually for each sample. Images were collected in 8-bits per pixel. All recordings were performed at room temperature (20–25°C). All image processing was performed with Zen imaging software (Zeiss) and ImageJ (W. Rasband, National Institutes of Health).

### Micromechanical Characterization of the MSC Surface

Micro-indentation microscopy experiments were carried out with a TI950 Triboindenter (Hysitron) coupled to an optical microscope to perform elastic moduli and hardness measurements of dry mature seeds after imaging scans. A conical tip capped with a 1 μm diameter hemisphere was chosen based on the need to perform measurements at a precise location, i.e., the top of radial cell walls. The first step consisted in capturing a picture of the seed surface by applying a 20 μN force at each pixel of a 128 × 128 pixel (60 μm^2^) image. Using this imaging mode provides the topography of the seed surface. Secondly, radial and distal cell walls were located using either topography or a peak force error map, which is an image of the error made on the target maximum force at each indentation. For each specific targeted point (usually 10 for distal and 10 for radial locations), a 1,000 μN force was tested and applied for 5 s and released in the same amount of time, allowing a penetration into the sample of 300 nm, which corresponds approximately to the primary cell wall thickness.

Load–displacement curves were then analyzed with the Triboscan and R softwares. For the 1 μm conical tip, a shape calibration was carried out, yielding the contact area *A*(*d*_c_) as a function of contact depth, and used in the calculation of the elasticity moduli in GPa. Extraction of the elastic modulus (*E*) was based on the slope of the force–indentation retract curve *k* (stiffness in μN/nm) and the contact area *A* (Supplementary Figure S8C) (Oliver and Pharr, 2004). The force (*P*) vs. depth (*d*) curve was fitted with the non-linear form

(1) $$P=c{\left(d-d_{f}\right)}^{m}$$

between 30 and 90% of the maximum force, from which *k* was computed as the derivative of force with respect to depth at maximal depth *h*_m_. The contact depth was computed as *d*_c_ = *d*_m_ − $\frac{3}{4}$ *P*(*d*_m_)/*k*. The elastic modulus was deduced as:

(2) $$E=\frac{\sqrt{\text{π}}(1-v^{2})k}{2\sqrt{A(d_{c})}}$$

*A*(*d*_c_) being the area of the probe at depth *d*_c_. Estimation of the hardness was also based on the unloading process (Supplementary Figure S8D) following:

(3) $$H=\frac{P(d_{m})}{A(d_{c})}$$

R was used for the statistical analysis of the data (R Development Core Team, 2016). After checking the normality of the distribution using frequency histograms and Shapiro’s test assuming that a *p*-value lower than 0.1 is acceptable for normal distribution, we applied a one-way ANOVA test, considering a *p*-value smaller than 0.05 to yield significant differences.

### Environmental Scanning Electron Microscopy of the Seed Surface

Dry seeds were mounted directly on specific ESEM microscope stubs fitted on a Peltier stage working at 2°C in a Quanta 200 FEG high resolution environmental scanning electron microscope (ESEM; FEI company) under a variable water partial pressure. Images were recorded using the FEI imaging system with an accelerating voltage of 5 kV, at a working distance of 4 mm. In order to control precisely and follow the kinetics of the mucilage secretion process, three successive increase/decreases of the partial pressure in the chamber were applied on the seed. Step by step, the vacuum was adjusted from 3 Torrs where the seeds are considered in extremely dry conditions (30% relative humidity) up to 6 Torrs corresponding to the full seed imbibition in water (100% relative humidity and condensation). Image analysis for slight brightness and contrast improvement was carried out using ImageJ software (W. Rasband, National Institutes of Health).

### Secreted Mucilage Content Quantification and Composition Analysis

Mucilage extraction were performed by placing flax seeds in ultra-pure water at 25°C without mixing (2.5 mL H_2_O for 100 mg seeds). Three countercurrent extraction phases were run for 4 h (extraction 1), 4 h (extraction 2), and 16 h (extraction 3) (Ziolkovska, 2012; Paynel et al., 2013). After collection, secreted mucilage samples were combined, freeze-dried and weighed. This screen of mucilge content was done on seeds in the F12 generation of a flax RIL population, cultivated in greenhouse conditions.

An aliquot of each mucilage sample (2 mg) was hydrolysed for 4 h at 100°C with 1 mL of 4 M trifluoroacetic acid. Mucilage composition was determined using high pH anion-exchange chromatography (HPAEC) on a Carbopac PA-1 analytical column (2 mm × 250 mm) with an elution flow rate of 1 mL/min (eluent A, 16 mM NaOH; eluent B, 160 mM NaOH; eluent C, 160 mM NaOH + 600 mM AcONa). Neutral and uronic sugars were eluted successively, following a gradient of 100% of A for 18 min, 26 min of linear gradient of B from 100 to 30% and C from 0 to 70%, 2 min of linear gradient of B from 30 to 0% and C from 70 to 100%, 30%, 5 min of linear gradient from 0 to 100% of A, and 100% of A for 35 min for the equilibrium step. Detection and carbohydrate concentrations were determined according to Guilloux et al. (2009). The contents of RG I and AXG (arabinoxylans and xyloglucans) were estimated as Rha + GalA and [Ara + Xyl + Glu], respectively.

### Statistics

Statistical analyses were performed using one- or two-way ANOVA or non-parametric Kruskal–Wallis analysis according to experimental constraints using R and Prism (GraphPad^5^). For each statistical test, we performed appropriated multiple comparison *post hoc* test with *p*-values correction.

## Results

### Identification of Three Recombinant Inbred Lines (RILs) With Contrasting Released Mucilage Content Phenotypes

**FIGURE 1:**
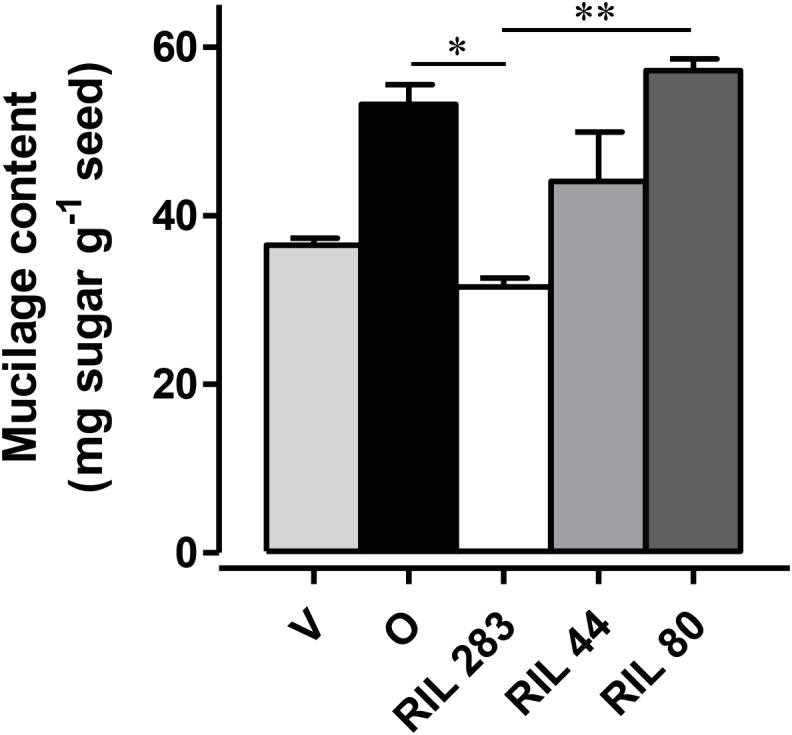
Phenotypic analysis to identify one reference and two contrasting released mucilage content candidates. The quantification of the total mucilage content extracted from seeds from plants grown in controlled conditions was achieved after 24 h of sequential water extraction of the mucilage. RIL 283 and RIL 80, respectively, release low and high mucilage content. RIL 44 was chosen for its intermediate phenotype. (*n* > 3, error bars denote SEM, ^∗^*P* < 0.05, ^∗∗^*P* < 0.01, Kruskal–Wallis *H*-test followed by a Dunn’s pairwise multiple comparison *post hoc* test with Bonferroni-corrected *p*-values). Bars = 4 mm.

This screen was done on a flax RIL population obtained by crossing Oliver (oil-flax) and Viking (fiber-flax) cultivars. These lines were analyzed for released mucilage content by using a 24-h sequential extraction with water (Ziolkovska, 2012; Paynel et al., 2013) on seeds in the F12 generation cultivated in greenhouse conditions. Two RILs were selected for severe mucilage content differences, i.e., RIL 283 – which has low mucilage content and RIL 80 – which has high mucilage content. RIL 44 was chosen as the reference RIL due to its intermediate phenotype close to both parental lines (Figure 1).

### Spatio-Temporal Specificities of Flax MSCs Structure and Mucilage Biosynthesis

To determine the cellular structure of the flax epidermal cells, an analysis of the flax MSCs structure during seed differentiation was performed on the RIL 44.

**FIGURE 2:**
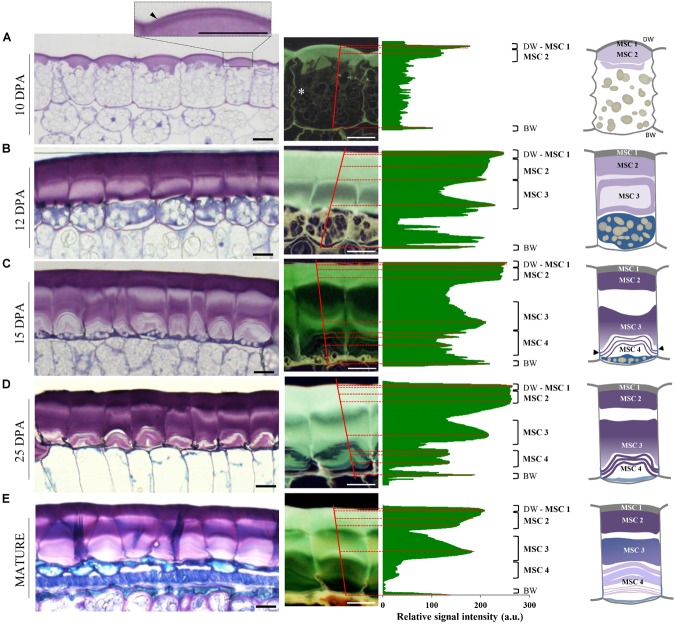
Flax mucilage is biosynthesized and organized in several successive layers inside MSCs. **(A–E)** Cytological analysis of flax seed coat sections from RIL 44 during seed development stained with toluidine blue O 0.01%. Plot profiles of the toluidine blue O relative signal intensity of representative mucilage secretory cells (right). Plot position corresponds to the red lines that span cells. Numbers on the right correspond to the mucilage layers observed, from MSC 1 to MSC 4. White asterisk in **(A)** shows the radial wall at 10 DPA. Before 10 DPA, the mucilage biosynthesis starts from the top of the cells in the apoplast, under the distal primary cell wall (dark arrowhead in **A**). **(B)** At 12 DPA, mucilage polysaccharide deposition continues until the middle of the cells. **(C)** At 15 DPA, while starch granules have been reduced to the bottom of the cells, the number of mucilage polysaccharides cell sublayers in MSC 4 has increased. **(D)** At 25 DPA, the mucilage polysaccharide cell layers appear unchanged. Note that the toluidine blue O staining intensity is darker in sublayers of MSC 4. **(E)** When seeds reach the mature stage, the number of mucilage polysaccharide sublayers has not changed in the upper part of the MSCs, but they are uncountable in the lower part. On the middle panels, the flax seed coat sections are presented during seed development by a relative signal that allow us to determine the different layers of flax MSCs. On the right panel, we present a schematic view of the organization of the four mucilage layers identified in this study and their polarized biosynthesis. Black arrowheads indicate radial wall reinforcement. BW, basal primary cell wall; DW, distal primary cell wall; MSC, mucilage secretory cell. Bars = 15 μm.

Observation of flax seed coat sections stained with toluidine blue O revealed that the deposition of mucilage polysaccharides starts in the apoplast at the upper face of MSCs before 10 days post-anthesis (DPA). This deposition occurs between the distal primary wall and the cytoplasm which contains a large number of starch granules (Figure 2A). From 10 DPA to the mature seed, starch granule depletion occurs, giving way to the formation of mucilage polysaccharide, arranged in a polarized manner from the top to the bottom of the cell (Figure 2A–E). From 12 to 15 DPA, the starch granules are reduced to a thin layer at the bottom of the MSCs and give way to a succession of several thin toluidine blue O-stained sublayers (Figure 2C; red dashed lines), separated by unstained sublayers. At 25 DPA, all starch granules had disappeared although no major structural differences were observed in MSCs, aside from sublayers at the bottom that appear to be strongly stained with toluidine blue (Figure 2D). At this developmental stage, mucilage biosynthesis is achieved; in flax, this process took twice as long as described in Arabidopsis (Beeckman et al., 2000; Western et al., 2000; North et al., 2014). The change in the toluidine blue O-staining intensity within each mucilage polysaccharide layer during seed development could result from a mucilage polysaccharide accumulation and/or a modification of the polysaccharide composition and/or different accessibilities. Taken together, these results precisely demonstrate the laminated structure of flax MSCs, composed of four major polysaccharidic layers (annotated from MSC 1 at the top to MSC 4 at the bottom of the cells in Figure 2; right panel).

### The Flax Seed Coat Mucilage Is Extruded Following a Peeling Mechanism Induced by Hydrated Mucilage Polysaccharides

**FIGURE 3:**
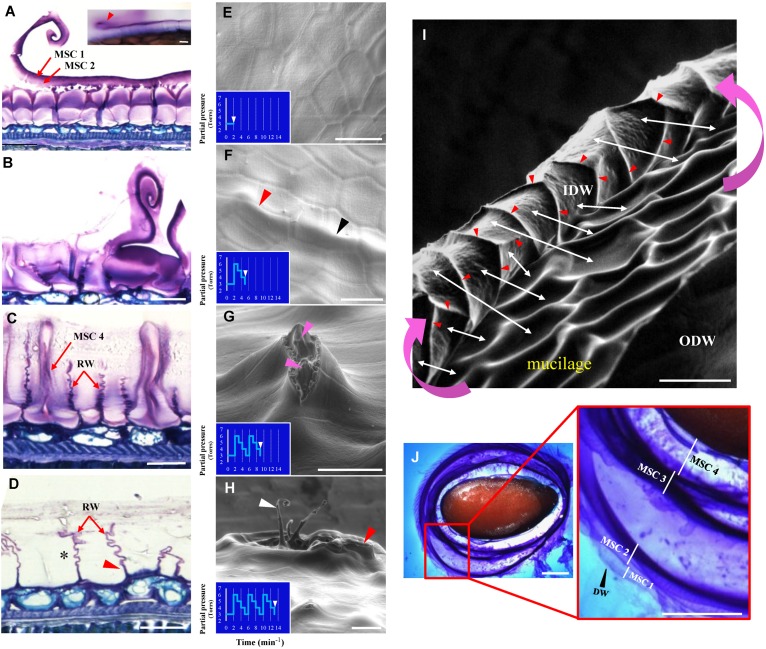
Flax seed mucilage release involves a peeling mechanism. **(A–D)** Cytological analysis of mature flax seed coat sections from RIL 44 stained with toluidine blue O 0.01% 24 h after imbibition. According to the degree of imbibition, each step of the mucilage release process can be observed. **(A)** Beginning of distal wall breaking and peeling. Picture in the top right corner shows the same pattern when whole seeds are soaked in water (red arrowhead). Note that MSC 1 and 2 (red arrows) are secreted along with the distal wall. **(B)** Advanced stage of the peeling process. **(C)** MSC 4 is being released. **(D)** Empty MSCs. Asterisk shows the “accordion” pattern of the radial cell walls. **(E–I)** Environmental scanning electron microscopy (ESEM) analysis of the mucilage release process submitted to successive increase/decrease water partial pressure steps. **(E)** Flax MSCs surface prior to water imbibition. **(F)** Characteristic profile of the flax MSCs surface after the first increase/decrease water partial pressure step. Domed surfaces can be observed both at the cell-to-cell junctions and in the middle of the cells (red and black arrowheads, respectively). **(G)** The second step enables the mucilage cell surface to break. Pink arrowheads show the mucilage polysaccharides. **(H)** The third step enables the fault to extend to neighboring cells and their distal cell walls to peel together. White arrow indicates the same peeling structures as observed in **(A)** and red arrow the same structure as observed in **(I)** from a different view angle. **(I)** SEM micrograph of the flax MSC peeling mechanism. Pink curved arrows show the direction of the peeling mechanism. White arrows indicate for some selected MSCs matches between the inner surfaces of their distal wall (IDW) and the inside of the MSCs. Red arrowheads show domed structures that delimit each MSC and correspond to the upper part of the radial walls. **(J)** The classical pattern of successive mucilage polysaccharides layers observed when mature flax seeds are soaked in water containing toluidine blue O 0.01%. IDW, inner surface of the distal primary cell wall; ODW, outer surface of the distal primary cell wall; RW, radial cell wall. Bars = 20 μm **(A–I)** and 1 mm **(J)**.

Based upon structural differences between Arabidopsis and flax MSCs, it can be hypothesized that the flax mucilage release process may occur differently to that observed in Arabidopsis (Beeckman et al., 2000; Western et al., 2000; Windsor et al., 2000). A cytological analysis of RIL 44 MSCs was performed after seed water imbibition (Figure 3A–D). The results indicate that the area under the distal primary cell wall breaks in some MSCs (Figure 3A), creating a fracture that extends over other cells allowing the distal part of the MSC to be released from one MSC to a next one according to a so-called “peeling” mechanism (Figure 3A,B). MSC layers 1 and 2 stay attached to the distal wall and are released with it. The same process can be observed when seeds are directly dipped in water containing toluidine blue O (Figure 3A; top right). Observation of the outer and inner surfaces of the MSC distal wall revealed a honeycomb structure (Supplementary Figures S1B,C). Once the MSC layers 1 and 2 are released, the other mucilage polysaccharide layers, i.e., MSC 3 and MSC 4, are successively released, resulting in empty MSCs (Figure 3C,D).

To examine whether the “peeling” mechanism originated from a pressure exerted by hydrated mucilage that expands into the MSC as suggested in Arabidopsis (Western et al., 2000; Macquet et al., 2007b; North et al., 2014), an experimental procedure was designed allowing the imaging of seeds using an ESEM while seeds are submitted to successive increases/decreases of water partial pressure (Figure 3E–I). The first step, which corresponds to a water partial pressure of 6 Torrs, generates bulges at the seed surface in localized areas, both at the cell-to-cell junction and in the middle of the cells (Figure 3F; respectively, red and black arrowheads). The second step allows the distal wall at the top of the domed cells to break (Figure 3G) and mucilage polysaccharides start to be released. Finally, the third variation of water partial pressure allows the fault to extend to neighboring cells and their distal walls to peel together (Figure 3H,I). These observations are consistent with the mucilage behavior upon hydration, which is released uniformly around the seed, when seeds are soaked in water containing toluidine blue O (Figure 3F) or ruthenium red (Supplementary Figure S2). This “multilayer” pattern of the mucilage release also confirms the laminated structure of flax MSCs and that the distal walls of neighboring MSCs peel together (Figure 3). These results demonstrate that pressure, probably from hydrated mucilage polysaccharides, induces the mucilage release process in flax seed.

### Rhamnogalacturonans I, Arabinoxylans, and Xyloglucans Are Not Located in the Same MSC Layers

**FIGURE 4:**
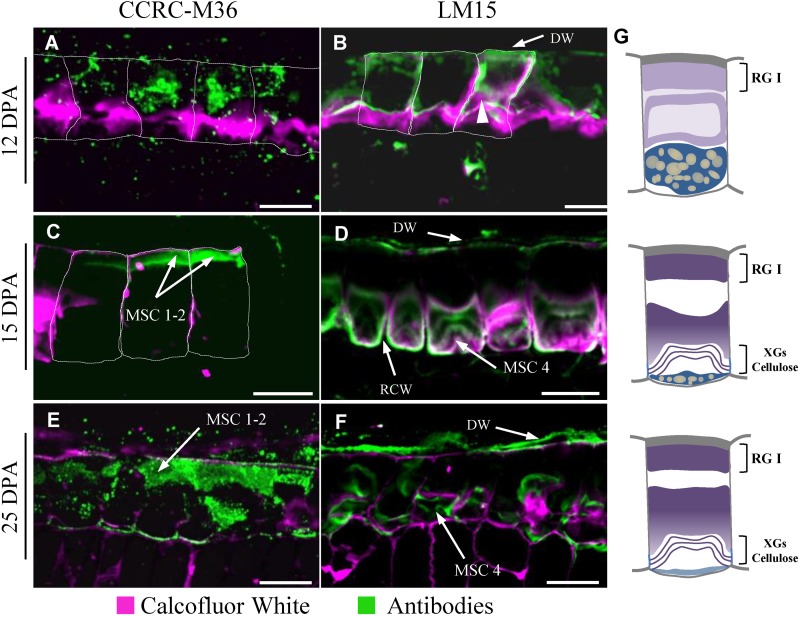
Polarized distribution of rhamnogalacturonan I and xyloglucans in flax MSCs. Seed coat sections from RIL 44 at 12 DPA **(A,B)**, 15 DPA **(C,D)** and 25 DPA **(E,F)** labeled with CCRC-M36 **(A,C,E)** and LM15 **(B,D,F)** antibodies recognizing unbranched RG I and xyloglucan epitopes, respectively. Calcofluor White, which stains both cellulose and other β-1,4-glycans, was used to visualize the cell walls. The outlines of the MSCs were manually drawn (white dashed lines) since distal and radial walls labeling are too weak, especially at 12 and 15 DPA. **(G)** Schematic view of transversal flax seed coat sections at 12 DPA (top panel), 15 DPA (middle panel) and 25 DPA (bottom panel). The putative chemical composition is reported in each MSC layer. DW, distal primary cell wall; RCW, radial cell wall. Bars = 40 μm.

Flax mucilage is composed of RG I, AXs, and probably HGAs (Warrand et al., 2005a,b; Naran et al., 2008; Paynel et al., 2013; Ray et al., 2013) and is distributed as multiple layers inside the MSC (Figure 2). This raises the question of whether the different flax mucilage polysaccharides are mixed together inside the MSC or are accumulated precisely in specific MSCs layers (or sub-cellular regions). An analysis of RIL 44 seed coat sections stained with toluidine blue O and ruthenium red, suggests that pectins such as RG I seem to be located in the upper face of the MSCs, i.e., MSC layers 1 and 2, whereas MSC 3 and 4 are more likely to contain neutral polysaccharides such as AXs and/or XGs (Supplementary Figures S3A,B). To confirm these observations, an immunohistological study of RIL 44 seed coat sections during seed differentiation was performed using the monoclonal primary antibody CCRC-M36 which binds to mucilage unsubstituted RG I (McCartney et al., 2005). At 12 DPA, CCRC-M36 binds mucilage in the upper face of the MSCs (Figure 4A). The punctuated pattern suggests that unsubstituted RG I is in the presence of other compounds or are not fully synthetized or are masked. From 15 to 25 DPA, CCRC-M36 strongly binds to MSC 1 and 2 (Figure 4C,E). These results are consistent with the strong CCRC-M36 labeling within the Arabidopsis mucilage ring. At 7 DPA (height of mucilage production), the mucilage pockets of Arabidopsis MSCs were brightly labeled with punctuate structures observed in the cytosol like in flax mucilage at 12 DPA. At 9 DPA, like in flax at 15 to 25 DPA, labeling of the mature mucilage pockets was strong, corresponding to the shift of polysaccharide production for mucilage to secondary cell wall synthesis (Young et al., 2008). This could be concomitant with the end of the unsubstituted RG I synthesis. Flax seed coat sections seem to be labeled with LM15 antibody, which binds primary cell walls and XGs (recognized the XXXG motif of XG; Marcus et al., 2008). At 12 DPA, XG antibodies bind primary cell walls whereas a thin labeling in MSC 4 starts to appear (Figure 4B). From 15 to 25 DPA, LM15 appears to label XGs within MSC 4 (Figure 4D,F). CCRC-M36 and LM15 labeling were confirmed on RIL 283 and RIL 80 (Supplementary Figure S4). These results were also confirmed with the monoclonal antibody CCRC-M58, which binds XGs with xylose substitutions (recognized XLLG; Supplementary Figure S5A) (Pattathil et al., 2010; Moneo-Sanchez et al., 2019). Finally, the monoclonal antibody LM11 was used to label AXs (Marcus et al., 2008). No labeling was detected within MSCs, while LM11 strongly labeled the cell walls of the sclerite cell layer from 15 to 25 DPA (Supplementary Figure S6). The absence of labeling of AX with the monoclonal antibody LM11 could be due to an epitope masked or simply not accessible.

### The Mucilage Release Efficiency Is Improved in RIL 80

**FIGURE 5:**
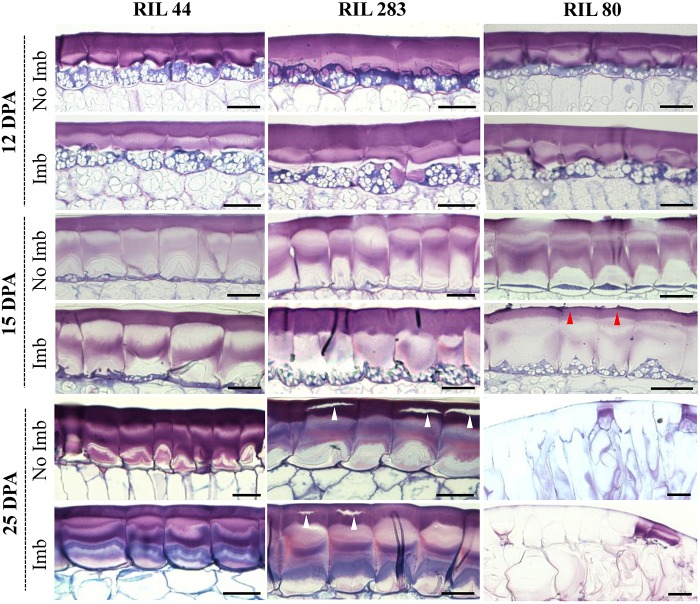
MSCs of RIL 80 are more sensitive to water imbibition relative to RIL 44 and 283. Structural and developmental analysis of seed coat MSCs from RILs 44, 283, and 80. Each image represent toluidine blue O-stained sections through the seed coat of intact seeds harvested at 12 DPA, 15 DPA, and 25 DPA. The time lapse of significant mucilage release is faster in RIL 80 compared to RIL 44 and 283 (red arrow), and in RIL 283 compared to RIL 44 (white arrow). For each developmental stage, seeds were analyzed before and 24 h after imbibition. Imb, imbibed; No Imb, non-imbibed. Bars = 40 μm.

Seed coat sections from RIL 44 and the selected RILs 283 and 80 were examined by cytological analysis before and after 24 h of seed water imbibition (Figure 5). At 12 DPA, no major differences were observed regarding the MSCs structure between the three RILs. At 15 DPA, the RG I-containing layers, i.e., MSC 1 and 2, appear thinner and less dense in the MSCs of RIL 80 when compared to other RILs, especially for water-imbibed seeds (red arrowheads). At 25 DPA, MSCs of RILs 44 and 283 do not show major differences according to their structure and do not release mucilage with or without seed water imbibition. On the contrary, RIL 80 releases its mucilage in the surrounding medium in both experimental conditions (Figure 5; right panel). These results suggest that MSCs of RIL 80 behave differently and release mucilage more easily at this development stage. Some cracks can be observed at 25 DPA on RIL 283 seed coat sections, suggesting a beginning of mucilage release. On the other hand, a mathematical modeling study revealed that mucilage expansion takes about 5 s (Deng et al., 2012). This approximately corresponds to the time lapse of the mucilage release (Supplementary Figure S2). By placing seeds from the three RILs in water containing toluidine blue O, we confirmed that the time lapse of significant mucilage release is faster in RIL 80 compared to RIL 44 and 283, and in RIL 283 compared to RIL 44 (Supplementary Figure S7; white arrowheads).

### Mechanical Forces Originate From Inside MSCs

**FIGURE 6:**
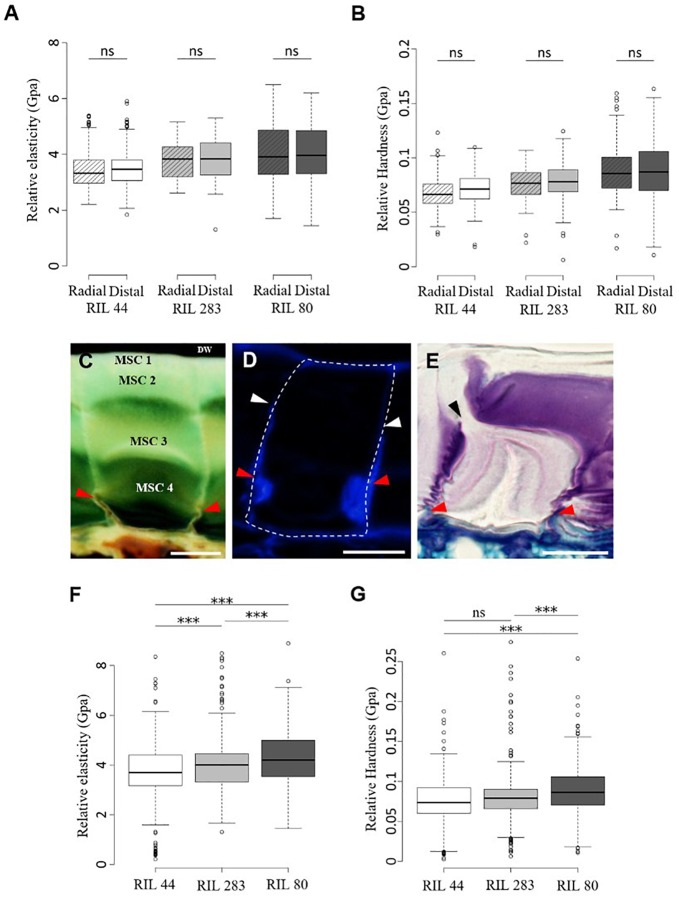
Mechanical forces that mediate mucilage release originate from inside MSCs. **(A,B)** Micro-indentation analysis of the rheological properties of the dried mature flax seed surface. Measurements of the relative elasticity (elastic moduli) **(A)** and hardness **(B)** were performed at the radial and distal walls surface in RIL 44 and selected RILs. **(C–E)** Cytological analysis of the radial wall structure of flax MSCs performed on RIL 44. Red arrowheads show structural reinforcement at the bottom of the radial walls. **(C,E)** Toluidine blue O-staining of flax seed coat sections. **(D)** Calcofluor White labeling of flax MSCs seems to reveal a secondary cell wall reinforcement that stops under MSC 2 (white arrowheads). **(E)** Picture illustrating the radial wall breaking point under MSC 2 (black arrowhead). **(F,G)** Measurements of the elastic moduli **(F)** and hardness **(G)** in RIL 44 and selected RILs combining data from radial and distal walls. Values are means of thirteen biological replicates with twenty measurements on each (*n* = 260 measurements per RIL, error bars denote SD, ^∗∗∗^*P* < 0.001, one-way ANOVA followed by a *post hoc* Tukey’s HSD). DW, distal primary cell wall. ns, not significant. Bars = 20 μm.

Micro-indentation experiments were performed on the distal and radial walls of the outer surface of intact dry seeds for the three selected RILs, i.e., in the middle of MSCs’ surface or at the junction between two MSCs (Supplementary Figures S8A,B), in order to measure the elasticity and hardness properties (Figure 6A,B and Supplementary Figures S8C,D). The purpose of these experiments is to identify whether mechanical differences at the seed surface correlate with the distal cell wall breaking point allowing mucilage release. No significant differences were observed (Figure 6A, 7B). These results do not reveal specific weak points at the surface of MSCs. To examine the inside of MSCs, a detailed cytological analysis of seed coat sections was done using both toluidine blue O, which does not label callose (O’Brien et al., 1964), and Calcofluor White, which labels cellulose and other β-glycans within the MSCs (Windsor et al., 2000; Willats et al., 2001; Macquet et al., 2007a; Mendu et al., 2011). The results seem to reveal significant cellulosic-based secondary cell wall reinforcement at the bottom of the MSC radial walls (Figure 6C–E; red arrowheads). Calcofluor White stainings also appears to show that the radial wall reinforcement stops just under MSC 2 (Figure 6D; white arrowheads). In-depth observations of sections made on seeds soaked for 24 h in water and stained with toluidine blue O allowed the observation of breaking MSCs and the localization of the breaking point on the radial wall between MSC 2 and 3 layers (Figure 6E; black arrowhead). Thus, the junction between MSC 2 and 3 represents a brittle area that enables mucilage release when it is submitted to a high internal pressure from expanding polysaccharides. Once the MSC’s surface is peeled, these results are consistent with the domed structures found on the inner surface of the outer walls (Figure 3I; red arrowheads).

It has been mathematically demonstrated on *Capsella bursa-pastoris* (shepherd’s purse) seeds that before mucilage release, the volume of mucilage polysaccharides in MSCs increases up to 75-fold, which impacts the volume and surface of the seed (Deng et al., 2012). This indirectly suggests that the capacity of the MSC’s surface cell wall to expand or break under high pressure will determine the timing between the swelling of the MSCs and the mucilage release. Our ESEM micrographs of the flax seed surface when seeds are slightly soaked (6 Torrs) confirmed a domed surface on each MSC (Supplementary Figure S1A). Moreover, data from micro-indentation analysis on both radial and distal walls were combined to analyze the rheological properties of the seed surface of each RILs independently of the wall type. Our results revealed that the outer MSC surface of RIL 44 appears softer than in RIL 283, the latter also being softer than RIL 80 (Figure 6F). On the other hand, RIL 44 and 283 are less plastic when compared to RIL 80 (Figure 6G). No significant differences were observed between RIL 283 and RIL 44 with respect to the hardness measurements. Combined together, our results show that the MSC’s surface of RIL 80 appears different from the two other RILs, as previously observed with our phenotypic and cytological analyses (Figure 5). Since the MSC’s surface of RIL 80 is stiffer and more elastic compared to RILs 44 and 283, while easily releasing more mucilage, this suggests that the mucilage release process does not rely on the expansion of the surface cell wall. Instead, a line with a stiffer surface would reduce the bulging-out of MSCs, and consequently increase the load on inner radial walls. This would yield an earlier internal breakage, which may lead to an earlier release of mucilage in RIL 80 due to its stiffer surface walls.

### The Main Flax Mucilage Polysaccharides Ratio Plays a Key Role on the Mucilage Release Efficiency

The data presented above would suggest that the pressure inside MSCs is probably more important in RIL 80 since it contains and releases more mucilage. The breaking point within MSCs allowing mucilage release was determined between MSC 2, which contains RG I, and the layers below, which contain AXs and XGs. This suggests that the ratio between the main polysaccharides in flax MSCs could have a significant impact on the mucilage release efficiency.

**FIGURE 7:**
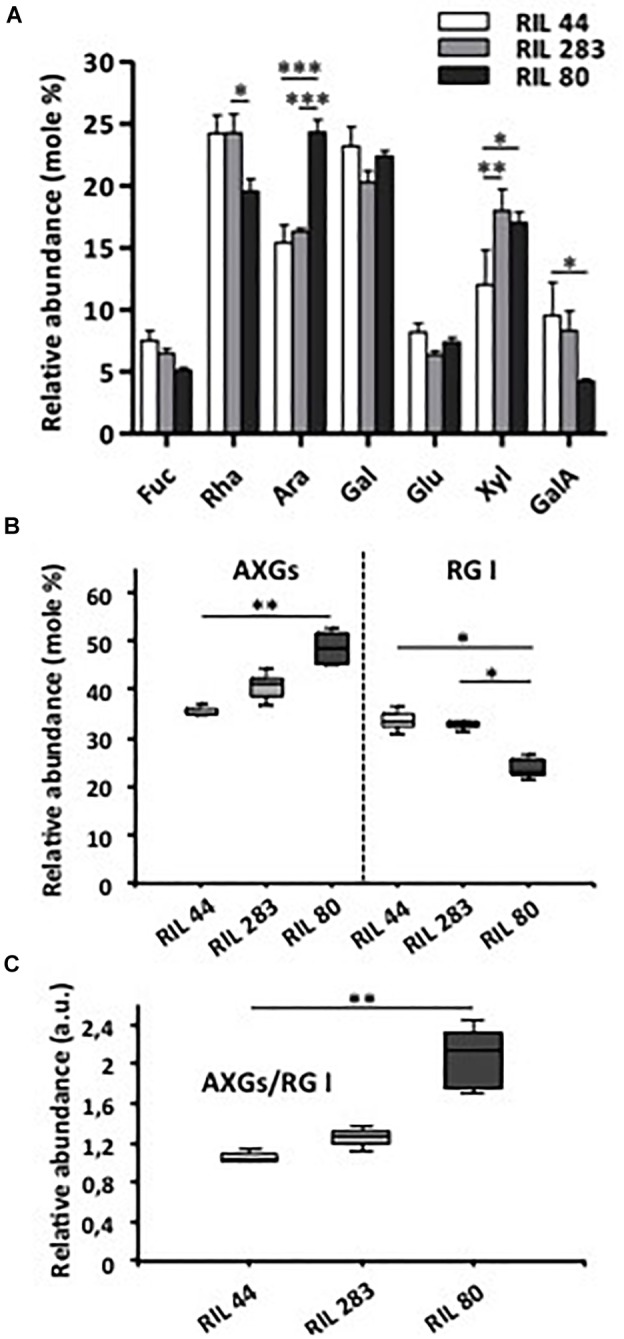
RIL 80 shows major differences in its mucilage chemical composition. **(A)** Monosaccharide composition of soluble mucilage extracts. Soluble mucilages were extracted from mature flax seeds of RIL 44 and both selected RILs 283 and 80 following three successive steps of 4 h, 4 h, and 16 h of water imbibition (Paynel et al., 2013). The monosaccharide composition analysis was performed using Anion Exchange and Size Exclusion Chromatography (HPAEC-PAD) (*n* > 3, error bars denote SEM, ^∗^*P* < 0.05, ^∗∗^*P* < 0.01, ^∗∗∗^*P* < 0.001, two-way ANOVA followed by a multiple comparison *post hoc* test with Bonferroni-corrected *p*-values). **(B)** Abundance of arabinoxylans, cellulose and xyloglucans (AXGs) and rhamnogalacturonan I (RG I) in mucilage extracts from mature seeds of the three selected RILs. Data in panel **(A)** were used to calculate the ratio AXGs/RG I in **(C)**. (*n* > 3, error bars denote SEM, ^∗^*P* < 0.05, ^∗∗^*P* < 0.01, Kruskal–Wallis *H*-test followed by a Dunn’s pairwise multiple comparison *post hoc* test with Bonferroni-corrected *p*-values).

We questioned if there were any variations in the mucilage chemical composition between the three selected RILs by completing an HPAEC-PAD analysis of the monosaccharide composition (Figure 7A). Our results reveal that the mucilage of RIL 80 shows major differences compared to the two other RILs, with less rhamnose and galacturonic-acid but more arabinose. We can also note that the amount of xylose is lower in RIL 44 compared to RILs 283 and 80. Because our cytological analysis showed the presence of RG I within MSC 1 and 2 and the presence of cellulose, β-1,4-glycans and XGs within MSC 4 (Figure 4 and Supplementary Figures S4, S5), we investigated whether the ratio of AXGs (Ara + Xyl + Glu) (Guilloux et al., 2009) to RG I (Rha + GalA) would have a significant impact on the mucilage release efficiency. Consistent with neutral and uronic sugars analysis (Figure 7A), RIL 80 contains less RG I and more AXGs compared to RILs 44 and 283 (Figure 7A). Moreover, the AXGs/RG I ratio is higher in RIL 80 compared to the other RILs (Figure 7B). We did not observe any statistically significant differences between RILs 44 and 283 for this ratio. Combined together, these results support the hypothesis that the easiest mucilage release observed in RIL 80 would be correlated with its higher AXGs/RG I ratio.

## Discussion

### The Flax Mucilage Polysaccharides Are Synthesized and Released Spatio-Temporally

**FIGURE 8:**
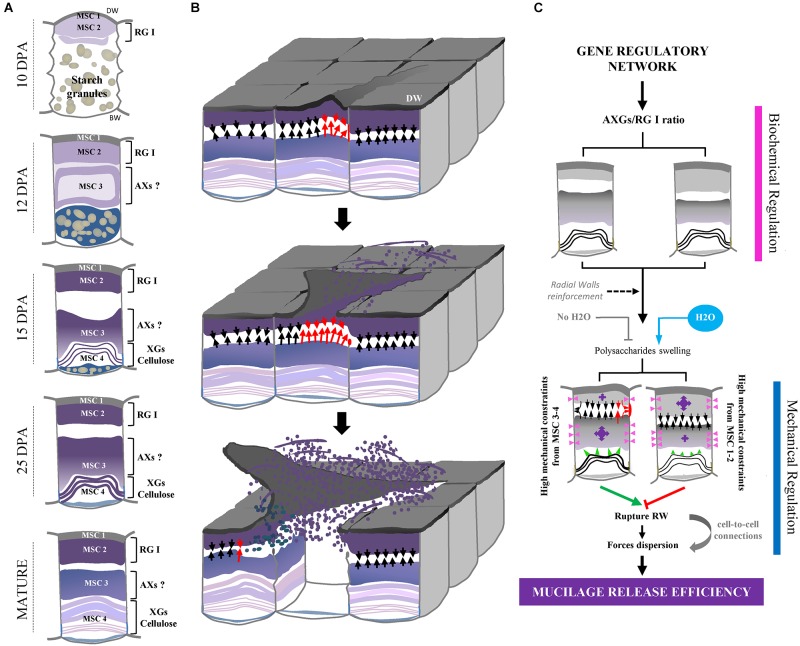
Overview of flax seed coat MSCs differentiation and of mucilage release process. **(A)** Schematic view of transversal flax seed coat sections during development. All the four MSC layers identified in this study and their corresponding putative chemical composition are reported. **(B)** Proposed overview of the flax mucilage release process when seeds are soaked in water, illustrated in 3-dimensions. Red arrows indicate the highest mechanical constraints exerted by MSC 2 on MSC 3-4 and conversely, which induces one of the two radial walls to break between MSC 2 and 3 and distal walls to peel. Then, the exerted mechanical pressure propagates and allows the mucilage release from other MSCs. **(C)** Proposed model relating the polysaccharide composition of flax mucilage and its release process. Purple arrows indicate the importance of the mechanical constraints exerted by MSC 2 on MSC 3-4 and conversely. Red rectangle indicates the breaking area. Arrows indicate high (red) and medium (black) putative mechanical pressures. Pink arrowheads also indicate putative mechanical forces from swelling polysaccharides in MSC 2 and 3 on radial walls. Since these forces come from expanding polysaccharides in MSC 2 and 3 of neighboring cells, the pressure applied on the corresponding radial walls is null. Green arrowheads show putative mechanical forces from MSC 4 on layers above. DW, distal primary cell wall.

In this study, we have first investigated the flax MSC’s structure and the mucilage synthesis during seed differentiation (Figure 8A). Our structural analysis revealed a link between the synthesis kinetics of the different MSC layers and their respective polysaccharide composition. The deposition of mucilage polysaccharides starts with MSC 1 and 2 from the top of the MSCs (Figure 2A,B), while RG I are detected within the same layers (Figure 4A,C,E and Supplementary Figure S4A). The layer MSC 4 appears subsequently, around 15 DPA at the bottom of the MSCs (Figure 2C), which corresponds to a significant labeling of XGs (Figure 4D). These results suggest that, in flax MSCs, RG I is synthesized first in the upper part of these cells, followed by XG synthesis at their bottom. Moreover, the honeycomb structure of flax MSCs could play a role in the adhesion of MSC layers 1 and 2 (Supplementary Figures S1B,C).

For the labeling of highly branched xylan in mucilage, additional antibodies like CCRC-M139, could be tested in future studies on flax seeds (Voiniciuc et al., 2015a).

The CCRC-M36 labeling in MSC 1 and 2 indicates the presence of unbranched RG I, even though RG I extracted from flax mucilage was demonstrated to be highly branched (Naran et al., 2008). This could explain why CCRC-M36 labeling shows a punctuated pattern (Figure 4A,C,E and Supplementary Figure S4A).

On the other hand, flax MSCs are also known to contain a high amount of AXs (Cui et al., 1994; Fedeniuk and Biliaderis, 1994; Warrand et al., 2003, 2005a,b; Paynel et al., 2013), although our cytological analysis does not reveal LM11 labeling within MSCs (Supplementary Figure S6). The same contradiction has been observed on Arabidopsis seed mucilage since AXs were found in the mucilage (Walker et al., 2011) whereas no labeling was observed using the LM10 monoclonal antibody (McCartney et al., 2005). In both cases, this could be explained by the differences in the degree of AX substitution that prevent LM11 binding to its epitopes. More specific antibodies would be required to elucidate this point. Moreover, flax mucilage does not only contain RG I and AXs, but a third fraction corresponding to an AX-RG I composite material (Naran et al., 2008). Since this composite could be based on the link between both polymers (Naran et al., 2008), it is conceivable that the punctuated pattern of CCRC-M36 as well as the lack of LM11 labeling might be due to the presence of this composite, which completely or partially prevents antibodies to bind. It is also possible that flax mucilage contains a specific AX structure which could explain the non-specificity of anti-Ax antibodies used in this study. From our comparative cytological and immunolabeling analysis (Figure 4 and Supplementary Figures S2, S4), we could hypothesize that a large fraction of AXs could be localized in MSC 3 (Figure 8A). Moreover, the monosaccharide composition of the RILs mucilage confirmed the presence of AXs in the MSCs in agreement with results previously published (Warrand et al., 2005a,b; Guilloux et al., 2009; Paynel et al., 2013; Pavlov et al., 2014).

This study reveals the high degree of complexity of the spatio-temporal organization of the mucilage polysaccharides within MSCs. This complexity was confirmed by the labeling of MSC 4 with the monoclonal antibody CCRC-M141, which is known to only bind to flax mucilage among 54 polysaccharides tested (Supplementary Figure S5B) (Pattathil et al., 2010). This complex developmental model also raises the question of the need for flax seeds to synthetize RG I at the top of the MSCs, before the other polysaccharides. It has been suggested in Arabidopsis that seeds are unable to release mucilage upon water hydration prior to complete polysaccharide synthesis and maturation (North et al., 2014). Our cytological analysis suggests that the polysaccharide maturation in flax MSCs can be achieved as early as 25 DPA since the mucilage can be released from RIL 80 seeds (Figure 7). An exciting hypothesis would be that RG I are synthesized first to prevent the mucilage from being released too early during seed development, and that the sufficient pressure required to break radial walls of the MSCs can only be reached when polysaccharides from MSCs 3 and 4 are synthetized. Arabidopsis developing seeds do not release mucilage largely because the seed coat epidermal cells are surrounded by a hydrophobic cuticle and have not undergone programmed cell death and desiccation (Voiniciuc et al., 2015a). Thus, analysis by transmission electron microscopy (TEM) or by histological, chemical analysis could reveal that RIL 80 would have a defective cuticle (Beeckman et al., 2000; Molina et al., 2008; Voiniciuc et al., 2015a).

Our ESEM analysis shows that mucilage release starts from the third step of water partial pressure increase/decrease steps (Figure 3E–H). This suggests that this sufficient partial pressure required for the mucilage release would be about 18 Torrs.

### The Efficiency of the Flax Mucilage Release Process Is Affected by Polysaccharide Composition

To date, the mucilage extrusion process is not fully understood in flax. In this study, our data suggest that the phenotypic differences observed between the three selected RILs in term of mucilage release could result from different polysaccharide composition (Figure 8B,C). Firstly, RIL 80 releases more mucilage and faster in comparison with RILs 44 and 283 (Figure 1, 5 and Supplementary Figure S7), which correlates with a higher mucilage content and a higher AXGs/RG I ratio (Figure 1, 7C). Secondly, RIL 80 exhibits a stiffer and more plastic outer MSC surface. Thus, the mucilage content and the speed of mucilage release seem to be positively correlated with the rheological properties at the MSC surface. However, this correlation does not hold when we compare RIL 44 with RIL 283. In this case, only the speed of mucilage release (Figure 5; white arrowheads and Supplementary Figure S7) and not the mucilage content correlates with the rheological properties at the MSC surface, since RIL 283 contains less mucilage compared to RIL 44 but shows a faster mucilage release, a higher AXGs/RG I ratio and a stiffer and more plastic outer MSC surface. Thus, the established consensus about the mucilage extrusion process which would be linked to mucilage content needs to be adjusted. A slightly more complex model is presented in Figure 8C. Once flax seeds are soaked in water, the mucilage polysaccharides expand in the MSCs due to their hydrophilic properties. Here, we propose that the swelling of the mucilage polysaccharides contained in MSC 2 and MSCs 3 to 4, i.e., RG I and XGs (probably AXs too), respectively, leads up to a contact between MSC 2 and MSC 3 (Figure 8B,C). The following polysaccharide expansion creates a mechanical pressure exerted from the MSC layer to the radial walls. Since these forces come from expanding polysaccharides in MSC 2 and 3 of neighboring cells, the pressure applied on the corresponding radial walls is null. Thus, mechanical forces could be redirected to the junction between MSC 2 and 3 creating the contraint responsible for the radial wall break (Figure 8B,C). The key role of mechanical forces exerted on radial walls is supported by the “waving” pattern of radial walls when MSCs are open or empty (Figure 3C,D, 6E). It is important to note that the rupture occurs only on one side of the MSC which corresponds to the starting point of the peeling mechanism and thus the mucilage release (Figure 8B,C). On the other hand, we have also demonstrated that the outer MSC’s surface in RIL 80, which contains less RG I, appears stiffer and more plastic (Figure 6F,G). This suggests that the rheological properties at the MSCs surface would be linked to the RG I content in MSC 2. This could also signify that a harder outer wall is less sensitive to pressure from expanding polysaccharides in MSC 2 and that mechanical forces from expanding RG I would propagate in the opposite direction, toward MSC 3, increasing constraints on the radial wall and facilitating mucilage release as observed in RIL 80. Thus, constraints on the radial wall between MSC 2 and MSC 3 are increased in RIL 80 (Figure 8C; left panel) compared to RILs 44 and 283 (Figure 8C; right panel). It is important to note that this model can only be viable once MSC layers 3 and 4 are synthesized. Combined together, our results show that, instead of the mucilage content, the AXGs/RG I ratio could affect the forces required to modulate the mucilage release efficiency (Figure 8C). Despite the flat flax seed topology, we imaged the three RILs using ESEM (Supplementary Figures S8A–D) and revealed that cell-to-cell connections do not exhibit the same pattern in RIL 80 compared to the RILs 44 and 283 (Supplementary Figure S9). This could represent another possible event that would affect mucilage release, especially the transfer of forces from MSC to MSC resulting in the peeling of the MSC’s surface (Figure 8B,C).

The functional role of the developmental regulator SEEDSTICK on the structural and mechanical properties of the Arabidopsis seed coat was confirmed by using notably nano-indentation experiments (Ezquer et al., 2016). In this study, the mucilage release is impaired in the *stk* mutant, which correlates with an increase of the epidermal cell wall stiffness and the Rha and GalA contents in the tightly attached mucilage. This seems to correlate with our results since the stiffness of the outer MSC surface positively correlates with the RG I content. However, we found opposite results with respect to the correlation between the mucilage release efficiency, the stiffness and the RG I content. This may come from the great differences in the outer seed coat topography between Arabidopsis and flax seeds as well as the differences in their mucilage polysaccharide composition and organization within MSCs. In Arabidopsis, the MSC’s structure and their outer wall topography are linked by the presence of a central secondary cell wall structure called columella. In flax, the presence of this columella is still discussed (Western, 2012; North et al., 2014). In this study, our immunochemical analyses revealed significant cellulose and β-glycans staining within MSC 4 using calcofluor white (Figure 4 and Supplementary Figures S4–S6), suggesting the presence of a stratified columella-like structure at the bottom of the MSCs in flax. Moreover, α-XG labeling of Arabidopsis seed coat sections showed the presence of XGs in developing secondary cell wall of the columella (Young et al., 2008), despite the fact the XGs are abundant in hemicellulose in primary walls rather than secondary walls. Intriguingly, we showed that XGs in flax MSCs are localized within MSC 4 between calcofluor white-stained MSC 4 layers (Supplementary Figure S4C), or partially colocalizing with the Calcofluor White labeling (Figure 4B,D and Supplementary Figure S5). This reinforces our hypothesis of a columella-like structure in flax MSCs. In this structure, XGs could be localized within a succession of secondary cell walls and sometimes crosslinked with cellulose, as already suggested by Paynel et al. (2013). It is tempting to speculate that the opposite effects of the RG I content and the outer MSCs surface stiffness on the mucilage release efficiency between Arabidopsis and flax species would be linked to the shape of their columellae.

### Flax MSCs as a New Model for the Study of the Mucilage Release Process

Even though Arabidopsis seed mucilage has been widely studied as a model to highlight plant cell wall metabolism and allowed to lay the foundations of mucilage polysaccharide biosynthesis, the use of MSC alternative models found in other plant species, like *Camelina sativa* or flax could be helpful to enrich this field of research (Western, 2012; North et al., 2014). However, basic data are lacking especially concerning the structural features of the MSCs on such models. The flax mucilage composition is already well established (Warrand et al., 2003; Naran et al., 2008). Moreover, we have deeply characterized in this study the flax MSC’s differentiation during seed development and the spatio-temporal properties of mucilage polysaccharide deposition. The large size of flax MSCs, the highly structured flax MSC architecture compared with Arabidopsis allowed us to study the key role of the polysaccharide composition in mucilage release efficiency. Thus, this work shows that the mucilage release process would be related to the composition in RG I, AX, and XG of the different layers of the MSCs, the synthesis of these polysaccharides occurring in successive steps. This parameter could be mentioned beyond the factors that affect the mucilage yields such as genotype, environmental conditions and extraction procedures (BeMiller, 1973; Diederichsen et al., 2006; Paynel et al., 2013). Finally, further chemical studies on flax MSCs, Arabidopsis natural variants and other crop species with similar internal MSC structure, like *Sinapis alba* (Esau, 1977) and *Plantago ovata* (Hyde, 1970; Tucker et al., 2017; Yu et al., 2017) could promise new discoveries. Recently, three distinct mucilage layers with highly branched AXs has been described for the mucilage of *Plantago ovata* (Yu et al., 2017). Indeed, the use of flax MSCs as a biological model could highly contribute to improving knowledge about mucilage polysaccharide biosynthesis, properties and metabolism as well as enhancing the industrial potential of mucilage as a biological substitute for chemicals in the cosmetic and pharmaceutical industries.

## Author Contributions

FMe and OVW conceived the research project. FMi, OVW, ND, EP, AB, PM, SG, CP, HD, KP, and FMe designed the experiments and interpreted the results. ND and AB carried out the micro-indentation experiments and analyzed the results. FF and LZ carried out immunolocalisations. LD carried out environmental scanning electron microscopy experiments. FMi carried out all experiments and data analysis except for micro-indentation, ESEM analysis and immunolocalisations and wrote the manuscript. KP and FMe supervised the writing.

## Conflict of Interest Statement

All authors declare that the research was conducted in the absence of any commercial or financial relationships that could be construed as a potential conflict of interest.

## Footnotes

1 http://www.nikon.fr

2 http://www.agarscientific.com

3 http://www.plantprobes.net

4 https://www.ccrc.uga.edu/mao/wallmab/Home/Home.php

5 http://www.graphpad.com
