# Supplementary material for: Cytological Approaches Combined With Chemical Analysis Reveals the Layered Nature of Flax Mucilage
Source: Front Plant Sci. 2019 Jun 21;10:684. doi: 10.3389/fpls.2019.00684 (PMC6598216; doi:10.3389/fpls.2019.00684)
Supplement: Supplementary file 1 [file Data_Sheet_1.PDF]

**Supplementary data I**

# Cytological approaches combined to chemical analysis reveals the layered nature of the flax mucilage

Fabien Miart, Françoise Fournet, Nelly Dubrulle, Emmanuel Petit, Hervé Demailly, Loic Dupont, Luciane Zabijak, Paulo Marcelo, Arezki Boudaoud, Christophe Pineau, Stéphanie Guénin, Olivier V. Wuytswinkel, François Mesnard, Karine Pageau

## Figure S1

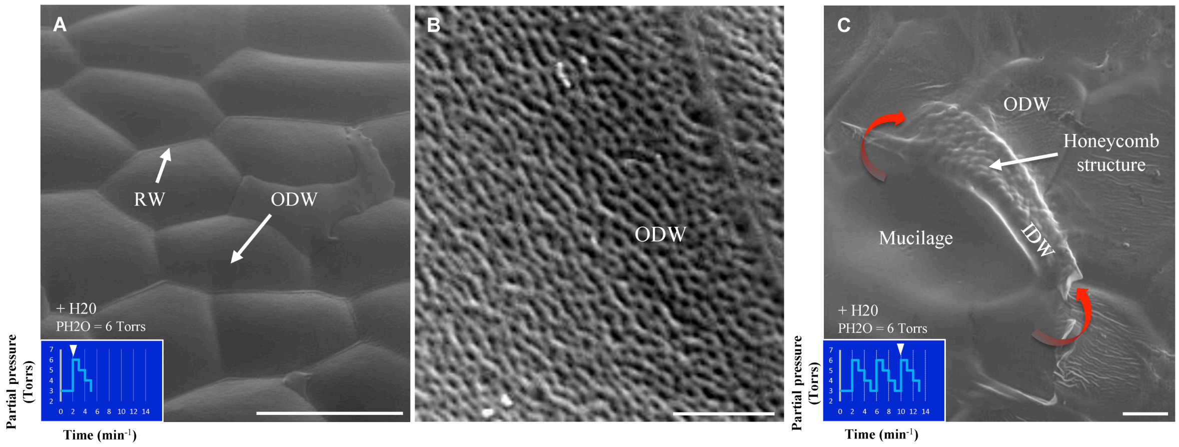


**Fig. S1.** Detailed structural analysis of the flax MSC inner and outer primary wall surfaces All experiments presented here were conducted under environmental scanning electron microscopy (ESEM). A, Flax seed surface topology during seed water imbibition showing the domed surface of each MSC. B, Study of the outer seed surface topology reveals a thin honeycomb structure. C, Once the distal wall detaches and peels due to the strong seed water imbibition (red arrows), the inner surface of the distal wall (IDW) can be observed with the good point of view and clearly shows an alveolar and scratchy surface. Each alveolus of the honeycomb structure is domed (white arrow) contrary to their hollow shape when observed from the outside. White arrowheads indicate water partial pressure status in the chamber. DW, Distal primary cell wall; RW, Radial cell wall. Bars = 50 $\mu$m (A), 3 $\mu$m (B) and 10 $\mu$m (C).

## Figure S2

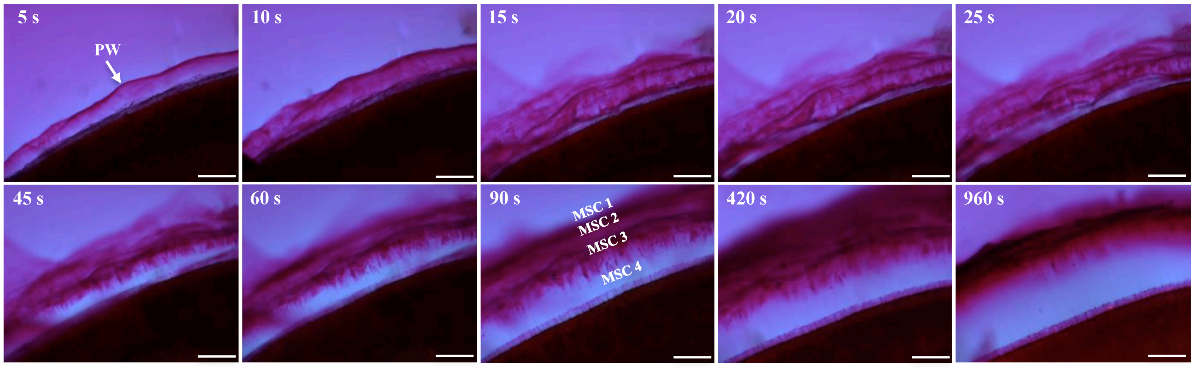


**Fig. S2.** Time lapse of the flax seed coat mucilage release mechanism
Mature flax seeds were placed in water containing ruthenium red 0.02 %. Note that the release mucilage process starts very quickly and the primary walls (PW) break almost instantaneously. The four mucilage polysaccharides layers from MSC 1 to MSC 4 can be observed at 90 s. Bars = 200 $\mu$m.

## Figure S3

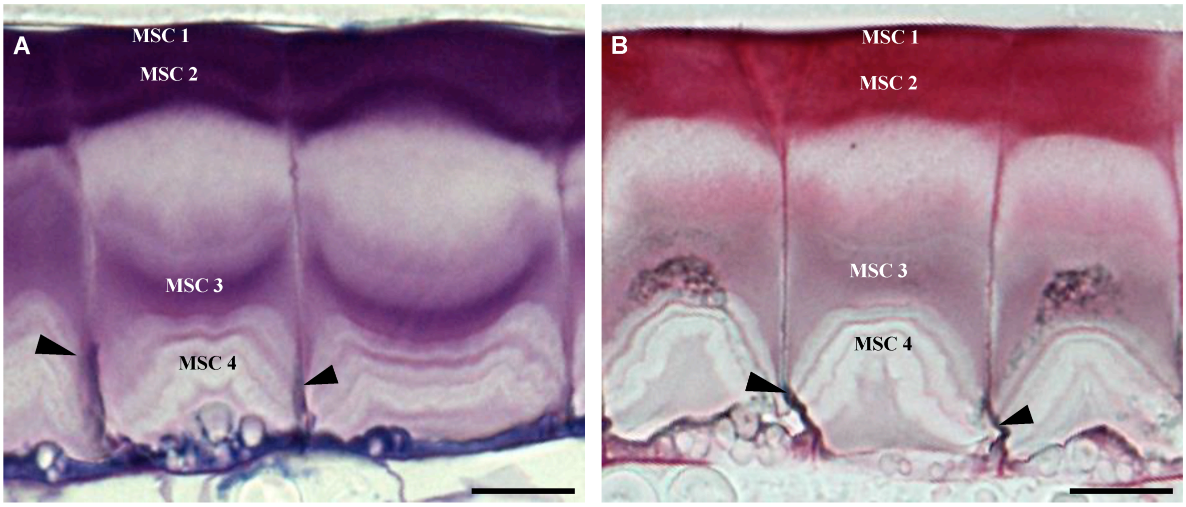


**Fig. S3.** Comparative cytological analysis of flax MSCs sections showing toluidine blue O and ruthenium red staining patterns
Seed coat sections were from seeds harvested at 15 DPA in order to obtain the best definition of the MSCs structure allowing to distinguish each mucilage polysaccharide layers and sublayers, especially within MSC 4. Seed coat sections were stained with toluidine blue O 0.01 % (A) or ruthenium red 0.02 % (B). Black arrowheads indicate radial walls reinforcement at the bottom of the MSC. Bars = 15 $\mu$m.

# Figure S4

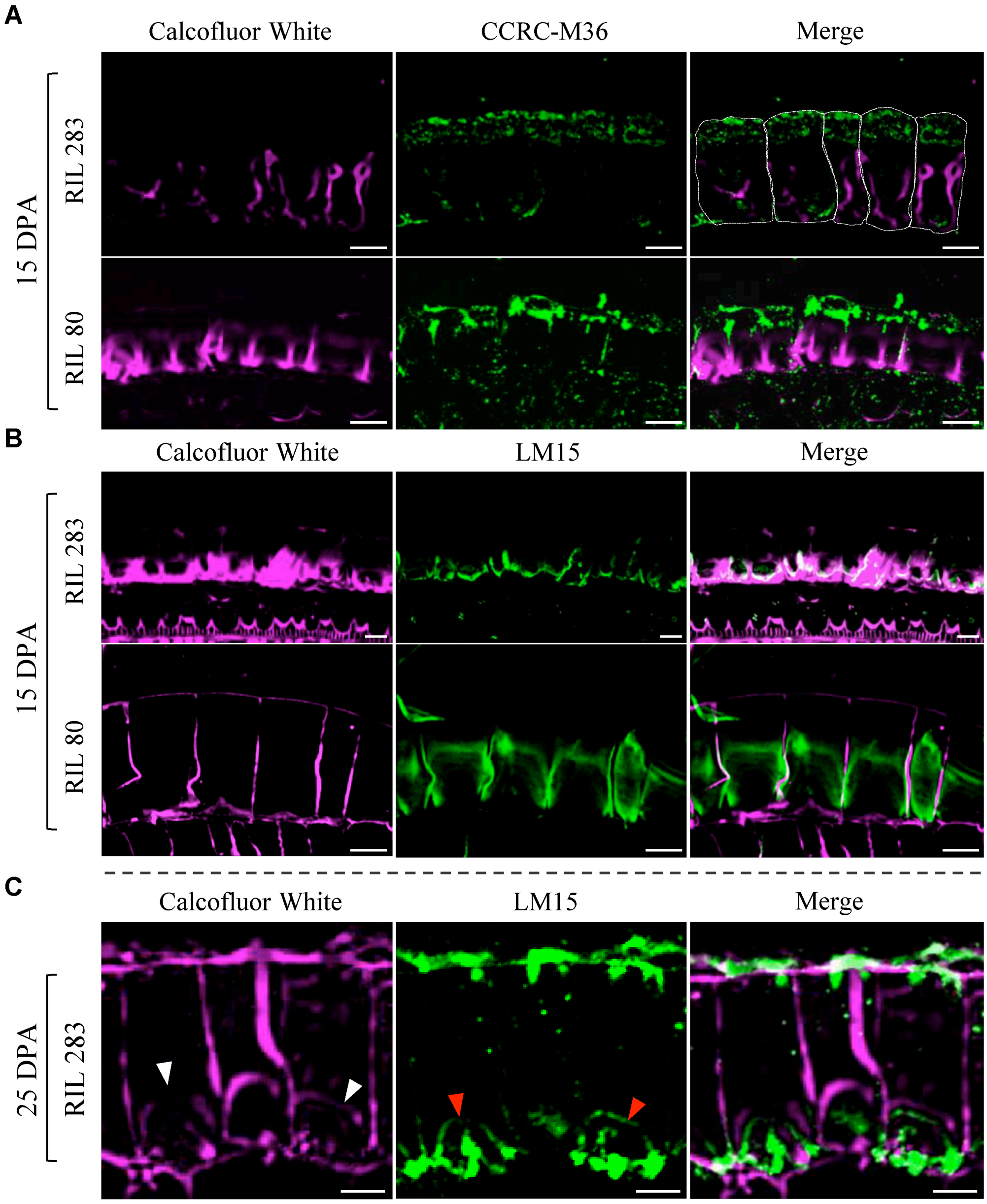


**Fig. S4.** LM15 and CCRC-M36 immunolabelings of RIL 283 and RIL 80 confirm and clarify XGs and RG I location within flax MSCs

All images show single sections of seeds at 15 DPA (A and B) or 25 DPA in C. A, Seed coat sections labeled with CCRC-M36. The outlines of the MSCs were manually drawn (white dashed lines) since distal and radial walls labeling are too weak. B, Seed coat sections labeled with LM15. C, Images are magnifications of seed coat MSCs from RIL 283 at 25 DPA showing alternation between LM15 and Calcofluor White-labeling within MSC 4 layer. Calcofluor White was used to visualize cell walls. Bars = 20 $\mu$m.
