# Supplementary material for: Cytological Approaches Combined With Chemical Analysis Reveals the Layered Nature of Flax Mucilage
Source: Front Plant Sci. 2019 Jun 21;10:684. doi: 10.3389/fpls.2019.00684 (PMC6598216; doi:10.3389/fpls.2019.00684)
Supplement: Supplementary file 2 [file Data_Sheet_2.PDF]

Supplementary data II

Cytological approaches combined to chemical analysis reveals the layered nature of the flax mucilage

Fabien Miart, Françoise Fournet, Nelly Dubrulle, Emmanuel Petit, Hervé Demailly, Loic Dupont, Luciane Zabijak, Paulo Marcelo, Arezki Boudaoud, Christophe Pineau, Stéphanie Guénin, Olivier V. Wuytswinkel, François Mesnard, Karine Pageau

Figure S5

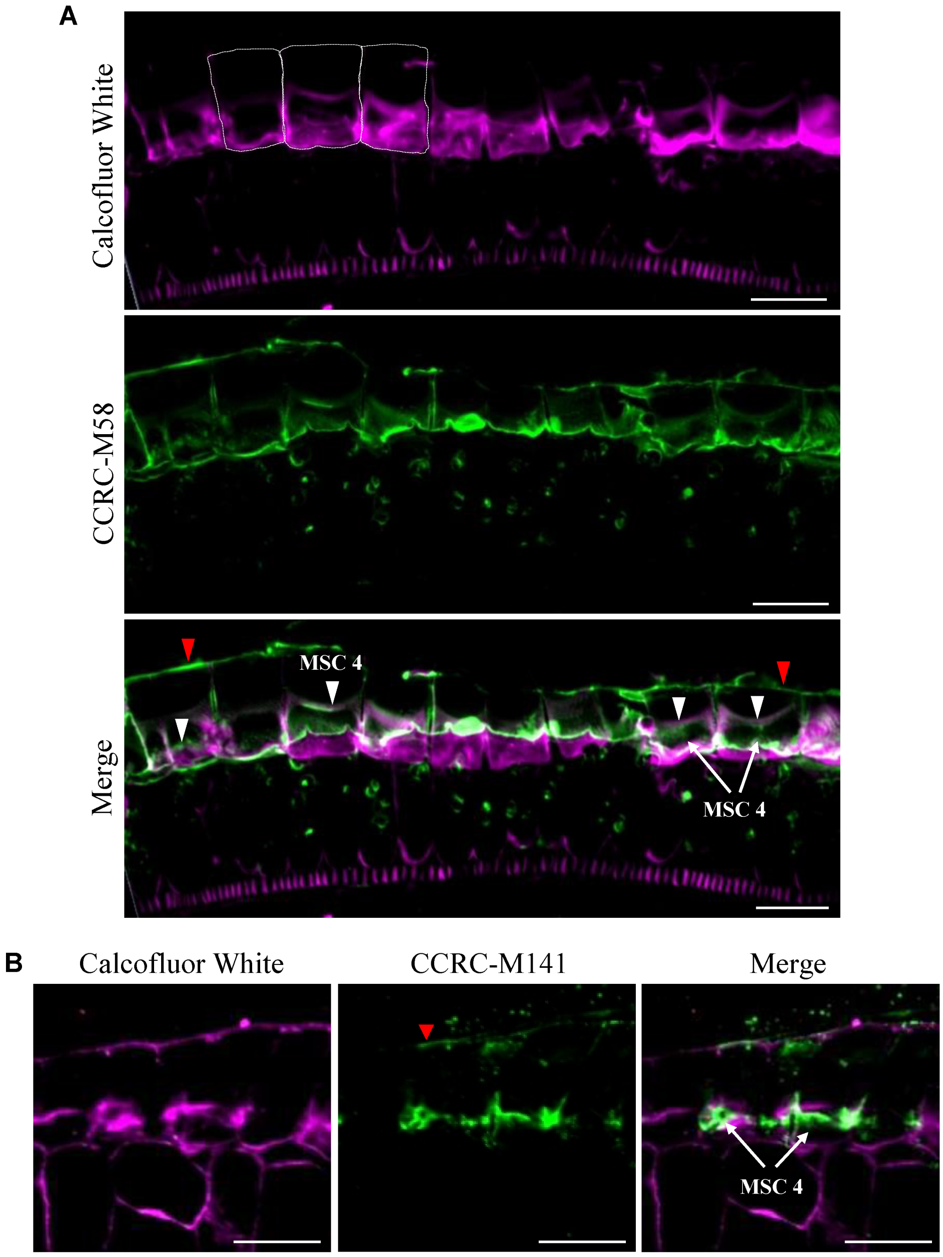

Fig. S5. CCRC-M58 and CCRC-M141 label mucilage polysaccharides in the bottom of MSCs

All images show single sections of seeds from RIL 44. A, Seed coat section at 15 DPA labeled with CCRC-M58 antibodies recognising xyloglucan epitopes. The outlines of the MSCs were manually drawn (white dashed lines) since distal and radial walls labeling are too weak. B, Seed coat section at 25 DPA labeled with highly specific CCRC-M141 antibodies only recognising flax mucilage polysaccharides. Calcofluor White, which stains both cellulose and β-1,4-glycans was used to visualize cell walls. Bars = 20 μm.

Figure S6

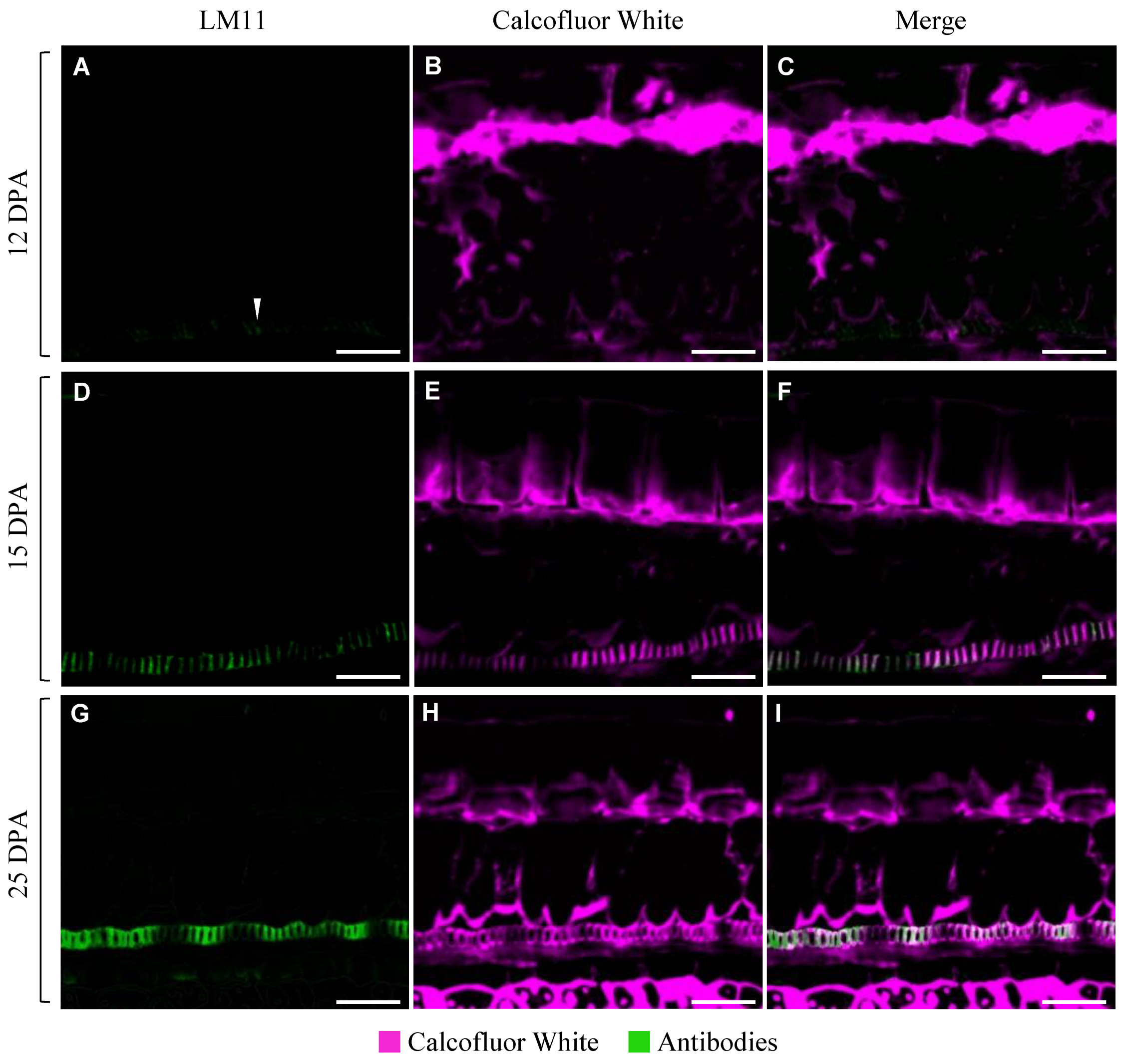

Fig. S6. LM11 does not label in flax MSCs but in the sclerite cell layer
All images show single sections of seeds from RIL 44 at 12 DPA (A to C), 15 DPA (D to F) and 25 DPA (G to I) labeled with LM11 recognising arabinoxylans (A, D and G) and Calcofluor White (B, E and H). Panel on the right corresponds to the merged images (C, F and I). Calcofluor White was used to visualize the cell walls. White arrowheads show LM11 labeling in the sclerite cell layer. Bars = 40 μm.
