# Supplementary material for: Cytological Approaches Combined With Chemical Analysis Reveals the Layered Nature of Flax Mucilage
Source: Front Plant Sci. 2019 Jun 21;10:684. doi: 10.3389/fpls.2019.00684 (PMC6598216; doi:10.3389/fpls.2019.00684)
Supplement: Supplementary file 3 [file Data_Sheet_3.PDF]

Supplementary data III

Cytological approaches combined to chemical analysis reveals the layered nature of the flax mucilage

Fabien Miart, Françoise Fournet, Nelly Dubrulle, Emmanuel Petit, Hervé Demailly, Loic Dupont, Luciane Zabijak, Paulo Marcelo, Arezki Boudaoud, Christophe Pineau, Stéphanie Guénin, Olivier V. Wuytswinkel, François Mesnard, Karine Pageau

Figure S7

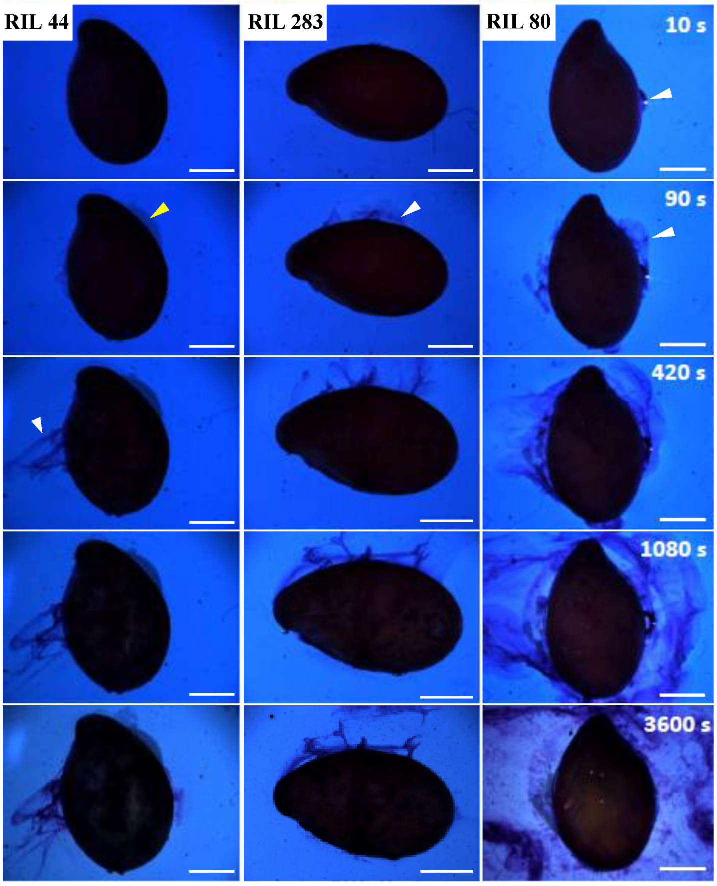

Fig. S7. Comparative kinetics analysis of the mucilage release in RIL 44 vs the selected RILs 283 and 80
Seeds were imbibed in water containing toluidine blue O 0.01 % and imaged from the start to the end of the mucilage release observed in RIL 80. White arrowheads indicate the significant starting point of the mucilage release in each RIL. Yellow arrowhead corresponds to the glue allowing to maintain the seed on the holder. Bars = 1 mm.

Figure S8

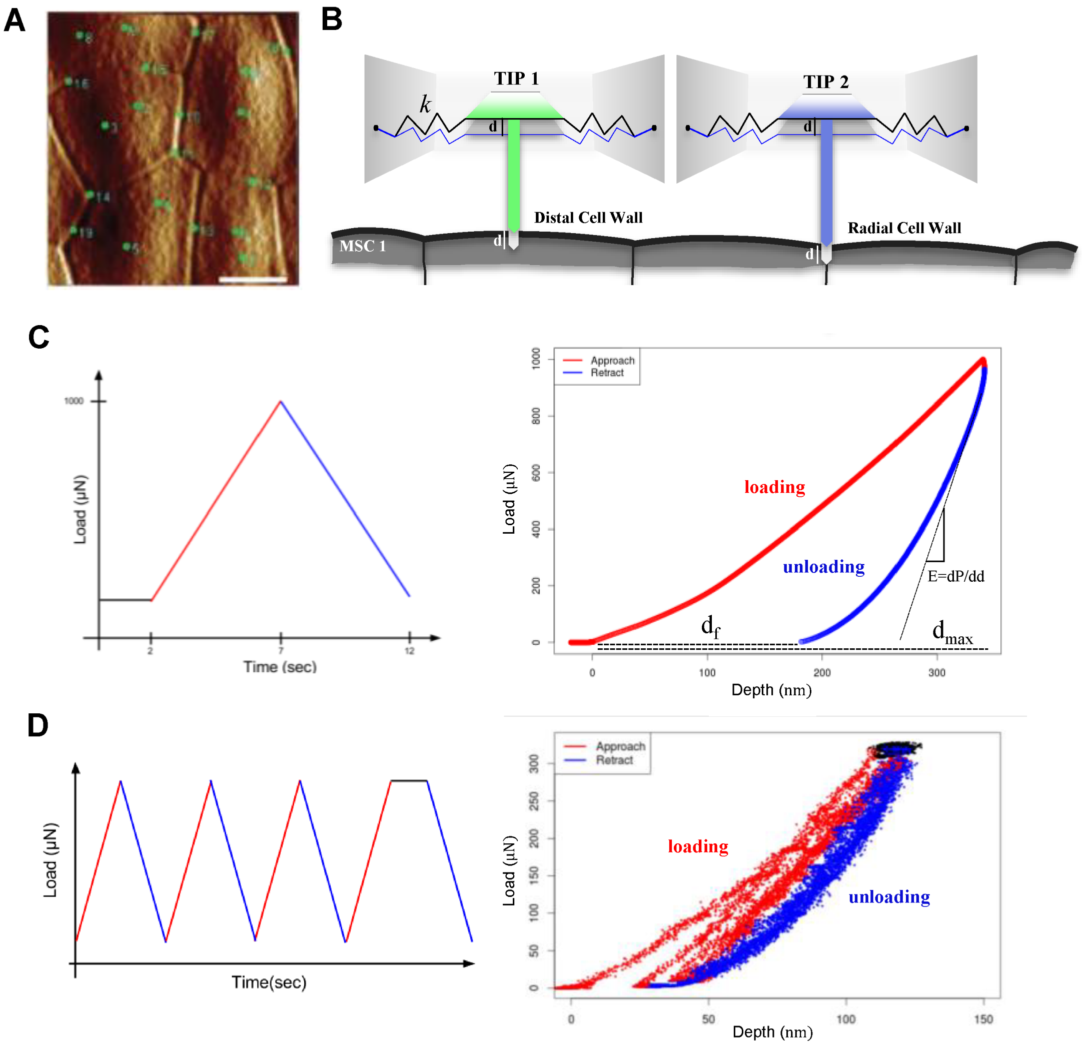

Fig. S8. Mechanical analysis of the flax MSC surface physical properties using micro-indentation microscopy in RIL 44 (full caption on next page)

A, Topographic image of the mature flax seed MSC surface and specific location of the micro-indentation targets on both distal and radial cell walls using the imaging mode. Note that the seed surface topology looks like the honeycomb structures observed by ESEM analysis in Supplemental Fig. 4B. B, Cartoon illustrating the principle of the micro-indentation methodology used to determine elasticity and plasticity differences between distal and radial walls at the seed surface. Following a scan of the seed, indentations were performed on the distal cell walls of MSC and on the top of MSC radial cell walls. Force (*k*) and tip displacement (d) are measured and recorded as soon as the tip is in contact with the sample. C, Desired indentation time and load (left) and example of indentation curve in the same order (right) for the elastic moduli measurements. Elastic modulus E was determined based upon the slope of the retract curve (blue) and the shape of the tip. Hardness H was determined based upon the maximum force and the shape of the tip. See the Materials and Methods section for details of the calculations. D, Desired indentation time and load (left) and

example of multiple indentation curves (right). Contact is reached for a larger depth at the second indentation, following many indentations, the extend and retract curves overlap suggesting that the tip « dug its hole » at the MSC surface. Taken together, these observations indicate irreversible plastic deformation of the MSC surface, which justifies the method used for the computation of elastic modulus and hardness. Measurements were performed on dried seeds. df, displacement in depth between the initial and final position of the tip (nm); dmax, maximal displacement in depth of the tip (nm). Bar = 15 μm.

Figure S9

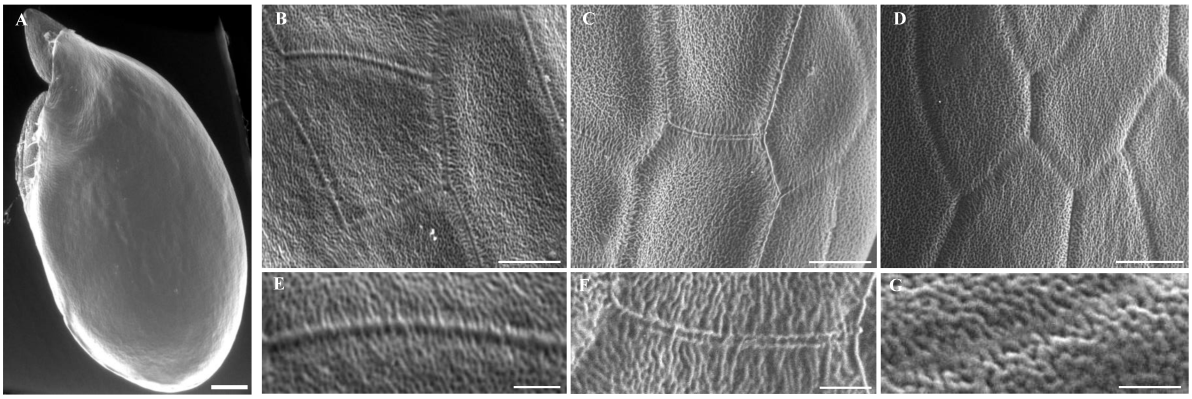

Fig. S9. Environmental scanning electron microscopy analysis of the flax seed surface from RIL 44 and the selected RILs

A, Micrograph illustrating the very flat whole flax seed topography. Different degrees of magnification were used to question the structural differences in RIL 44 vs the selected RILs, from the cellular (B to D) to the subcellular (E to G) levels. Bars = 500 μm (A), 10 μm (B-D) and 3 μm (E-G).
